# The Clinical Utility of Continuous Laryngoscopy During Exercise: A Report of Two Cases

**DOI:** 10.7759/cureus.50572

**Published:** 2023-12-15

**Authors:** Tomas Leng, Joshua Wiedermann, Shelagh Cofer, Sophia Pillai

**Affiliations:** 1 Department of Pediatrics and Adolescent Medicine, Mayo Clinic, Rochester, USA; 2 Department of Pediatric Otorhinolaryngology, Mayo Clinic, Rochester, USA; 3 Department of Pediatric Pulmonary Medicine, Mayo Clinic, Rochester, USA

**Keywords:** pediatric otolaryngology, pediatric pulmonology, exercise-induced laryngeal obstruction, continuous laryngoscopy during exercise, exertional dyspnea

## Abstract

Exertional dyspnea is a common and disabling symptom in otherwise healthy children and adolescents, as well as in children with baseline airway abnormalities. It impairs the quality of life and may be associated with fatigue and underperformance in sports. Exertional dyspnea can be caused by a wide variety of structural and psychogenic causes. Exercise-induced laryngeal obstruction (EILO) is a relatively prevalent entity in young people that usually presents with exertional stridor, coughing, and dyspnea caused by transient closure of the larynx. In more complex cases where conventional tests such as pulmonary function tests (PFTs), chest imaging, ECG, and echocardiography are unrevealing, continuous laryngoscopy during exercise (CLE) tests may provide diagnostic utility. In addition to the baseline abnormalities visualized by conventional laryngoscopy, CLE can assess dynamic laryngeal responses during exercise. This article describes the clinical characteristics of two pediatric patients with various degrees of laryngeal dysfunction at baseline and the utility of CLE testing in tailoring management strategies.

## Introduction

Continuous laryngoscopy during exercise (CLE) is a test that uses a flexible distal-chip laryngoscope secured on a head apparatus, and it is usually performed in conjunction with the cardiopulmonary exercise test (CPET) in a laboratory setting. In addition to the baseline abnormalities visualized by conventional laryngoscopy, CLE allows for the assessment of dynamic laryngeal responses during exercise. It provides real-time visualization of laryngeal movements during physical activity and has become the gold standard in diagnosing exercise-induced laryngeal obstruction (EILO) [1]. Exercise-induced laryngeal obstruction is a relatively prevalent entity in young people that usually presents with exertional stridor, coughing, and dyspnea caused by transient closure of the larynx [2-4]. The diagnosis of EILO is not straightforward because its features can overlap with exercise-induced asthma, which can result in inappropriate therapy. Several studies have suggested that EILO and asthma often coexist [5-7]. Exercise-induced laryngeal obstruction usually arises from supraglottic obstruction, although in some instances, it can result from inappropriate closure of the glottis, and a combination of both can occur [1].

Continuous laryngoscopy during exercise has been safely used across a wide range of ages, including the pediatric population, and it plays a pivotal role in guiding the management and follow-up of patients with EILO [8]. Speech therapy and various glottic and supraglottic surgical procedures have been incorporated into the management strategies of EILO. However, despite substantial technological advances, validated diagnostic and treatment algorithms have not yet been established [9-11]. Although the CLE procedure has been recognized as the diagnostic technique of choice for EILO, our clinical experience suggests that its utility reaches beyond this scope and can significantly influence therapeutic decisions.

## Case presentation

Case one

A 12-year-old boy with a complex medical history of prematurity, severe bronchopulmonary dysplasia, developmental delay, and prolonged mechanical ventilation presented with worsening exertional dyspnea. He had fixed obstruction of the airway at the supraglottic and glottic levels (arytenoid complex and immobile vocal cords) and underwent previous tracheal reconstructions at an outside institution. He had a history of persistent wet coughs and nighttime continuous positive airway pressure (CPAP) dependence. He presented for the evaluation of exertional stridor as well as a breathier and weaker voice.

**Figure 1:**
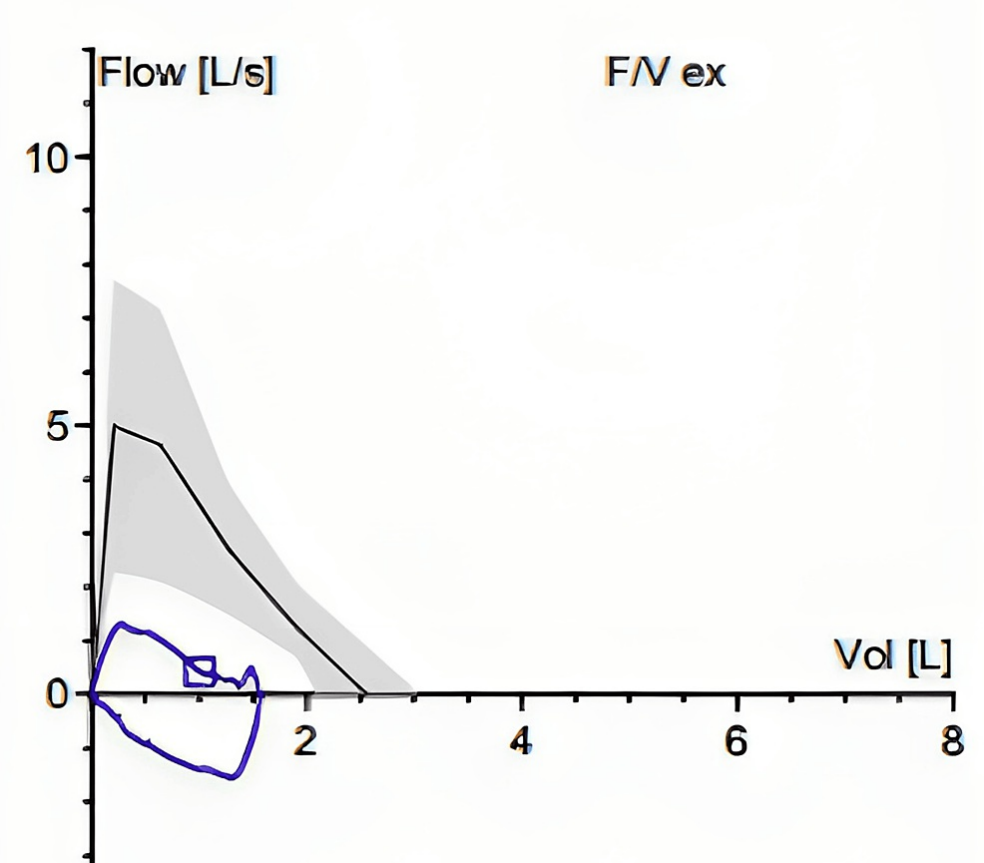
Flow volume (FV) loop consistent with a fixed upper airway obstruction The black line demonstrates a normal pattern of FV loop prediction based on age, height, and sex. The blue line is consistent with the flattening of the inspiratory and expiratory loops of the FV curve, indicating a fixed upper airway obstruction seen in our patient.

After considering the substantial risks of surgical interventions that could potentially worsen the voice/airway balance and a concern for dynamic EILO, he underwent a multidisciplinary team evaluation. Spirometry testing for baseline evaluation of lung function was limited due to poor technique; however, there was evidence of fixed airway obstruction as seen on the flow volume (FV) loop (Figure 1).

**Figure 2:**
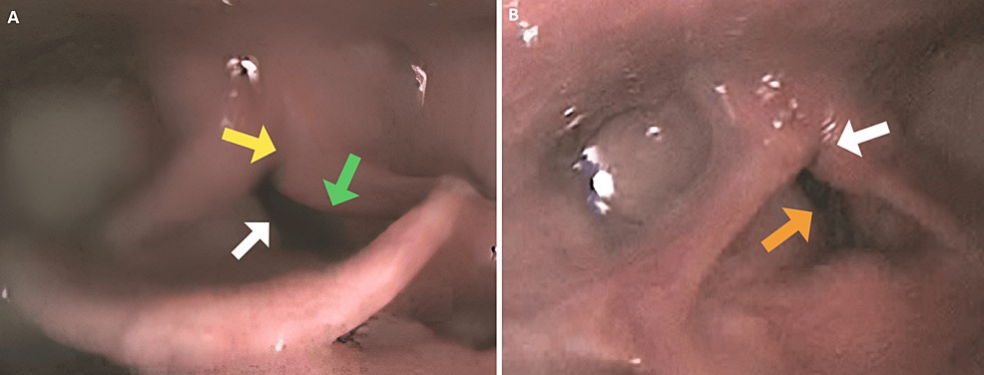
Continuous laryngoscopy during exercise (CLE) findings at rest and peak exercise A: CLE findings at rest: flattened laryngeal inlet with medialization of the vocal cords (white arrow), tethered arytenoid complexes (yellow arrow), and left arytenoid prolapse (green arrow). B: CLE findings at peak exercise: minimal to no movements of the vocal cords (orange arrow) and tethered arytenoid complexes obstructing the airway (white arrow).

Flexible and rigid bronchoscopy under sedation revealed supraglottic obstruction, mostly from the left arytenoid complex and epiglottic petiole prolapse, posterior glottic stenosis, subglottic stenosis, and tracheomalacia. As the perceived exercise symptoms were disproportionate to the already-known fixed airway obstruction, a decision to perform CPET with CLE was made. Continuous laryngoscopy during exercise confirmed fixed abnormalities of the upper airway. At rest, vocal folds appeared medialized with minimal abduction with inspiration (Figure 2A).

The laryngeal inlet appeared flat in the anterior-posterior dimension secondary to prolapse of the petiole of the epiglottis. Continuous laryngoscopy during exercise at peak exercise revealed the arytenoid complexes tethered together with minimal movements of the vocal cords (Figure 2B, Video 1).

**Video 1:** Case one: continuous laryngoscopy during exercise

The left arytenoid was prolapsing into the laryngeal inlet, obscuring one-third of the aperture with exertion.

There was a mismatch between ventilation demands and delivery through the narrowed laryngeal aperture, which caused the stridor to worsen as the exercise progressed.

Given these new findings on CLE, a thoughtful, step-wise, minimally invasive approach to surgical interventions was planned to optimize voice quality and improve exercise tolerance while minimizing the risk of aspiration.

Case two

An 11-year-old boy was evaluated for worsening exertional dyspnea and stridor. His medical history was significant for tracheomalacia, aortopexy, anxiety, mild asthma, and a type 2 laryngeal cleft repaired endoscopically using bilateral arytenoid flaps. There were no respiratory exacerbations or aspiration events, but he continued to experience stridor, dyspnea, and choking sensations in the airways. Symptoms slightly improved after adenotonsillectomy and arytenoid debulking.

**Figure 3:**
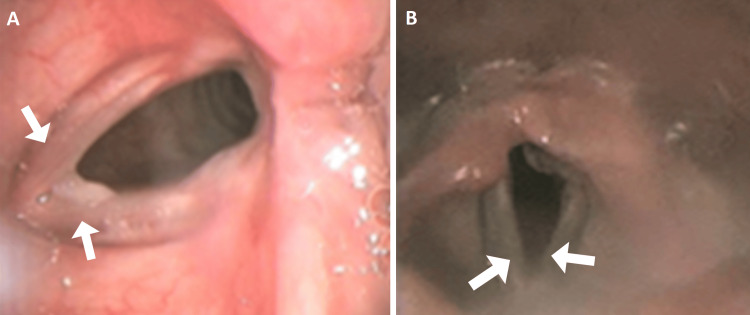
Continuous laryngoscopy during exercise (CLE) findings at rest and peak exercise A: CLE findings at rest (baseline): limited abduction of the vocal cords (white arrows) and tethering of the posterior glottis worse on the left. B: CLE findings at peak exercise: paradoxical adduction (incomplete) of the vocal cords (white arrows).

Pulmonary function tests (PFTs) showed mild obstruction without a response to bronchodilators. Laryngoscopy revealed symmetric vocal cord motion and a slight limitation of the abduction bilaterally. Due to the exertional nature of his symptoms, CPET with CLE was recommended to evaluate for additional dynamic EILO as a cause of his worsening symptoms. The results of his CPET with CLE showed borderline normal exercise capacity and limited abduction of the vocal cords with tethering of the posterior glottis at rest (Figure 3A).

A marked reduction in breathing reserve and respiratory responses during peak exercise, with clear evidence of paradoxical adduction of the vocal cords on CLE, was suggestive of a combination of anatomical and functional abnormalities (Figure 3B, Video 2).

**Video 2:** Case two: continuous laryngoscopy during exercise

The exaggerated heart rate response to the level of exercise was suggestive of an element of deconditioning. The paradoxical vocal fold movements were attributed to the panic attacks experienced with intense exercise activities, mostly due to the failure of compensatory ventilatory responses due to upper airway obstruction.

He took part in a program of graded exercise rehabilitation and speech therapy. At the follow-up visit after one year, he lost 25 pounds and his exercise symptoms resolved.

## Discussion

This report describes our experience of managing two cases of children with inducible laryngeal obstruction due to various etiologies. Information provided by CLE influenced our therapeutic decisions for both patients. Several reports on the diagnostic use of CPET with CLE in patients with EILO and asthma have been described [5, 12]. However, with regard to the utility beyond diagnostic capabilities and further therapeutic implications, reports are lacking. Since its introduction to clinical practice in 2006, CLE has become an essential tool in the diagnosis of patients with various degrees of functional laryngeal dysfunction.

Traditionally, options for evaluation were limited to rigid and flexible laryngoscopy and bronchoscopy. In contrast to these more conventional methods, CPET with CLE offers the benefit of direct visualization of laryngeal structures, with a focus on dynamic changes during different phases of physical activity. Dyspnea is a common symptom in both healthy children and children with surgical airways. In patients who underwent a procedure involving the upper airway, the perceived exertional symptoms may be caused by additional dynamic laryngeal responses resulting from the surgical alterations to the airway. Testing at rest, with methods such as PFTs and office laryngoscopies, does not always provide a complete picture. It can result in a delayed or incorrect diagnosis, negatively impacting treatment recommendations. Exercise testing using cardiopulmonary exercise has tremendous potential for determining exercise capacity and identifying ventilation abnormalities associated with exercise symptoms. A continuous video laryngoscopy assists in determining the cause of abnormal ventilation [1]. Olin et al. demonstrated that laryngeal obstruction is more severe during exertion at peak work capacity, submaximal exercise, and recovery than baseline obstruction present during rest [8]. In our report, CLE not only contributed to the identification of the underlying laryngeal pathology but also played a role in successful treatment alteration in both patients.

In patient one, CPET with CLE was chosen due to his worsening exertional dyspnea, as conventional endoscopy of the larynx did not identify pathology that would be responsible for the degree of exertional impairment seen in this patient. Continuous laryngoscopy during exercise allowed for a further assessment of laryngeal structures during exercise, allowing for the election of conservative stepwise intervention. In patient two, the initial endoscopic inspection revealed only a mildly reduced glottic aperture; however, paradoxical adduction of the vocal cords with forced inspiration and stridor was observed during exercise using CLE. In this patient, the use of CLE allowed for a non-surgical intervention that improved the patient’s symptoms. Overall, CLE was well tolerated in both cases, implying a favorable safety profile and suggesting that it could be used in large-scale studies. The primary reason for exercise termination was dyspnea in both patients. Baseline and exercise ECG demonstrated no evidence of ischemic changes in our patients. Heart rate and blood pressure responses were normal in case one, with an appropriate increase throughout the exercise. In case two, the heart rate response was mildly exaggerated for workload, suggesting deconditioning. No adverse events were reported, and both patients returned quickly to their baselines upon the termination of the study. A systematic review conducted by Thomader et al. reported that 10 (2.2%) out of the 455 subjects who underwent CLE experienced adverse events, including laryngeal spasm, procedural anxiety, hyperventilation attacks, vasovagal collapse during local anesthesia of the nose, and an asthma-like attack [5]. Although this proportion is not negligible, our experience and that of other centers suggest that the benefits outweigh the drawbacks, as information obtained during CLE can significantly alter treatment decisions [13]. While electromyography of the laryngeal muscles can objectively demonstrate paradoxical movement of the vocal cords, it requires a rather specialized technique and equipment, and CLE may be superior.

A study conducted by Hull et al. indicated that CLE offers a robust means of characterizing varying degrees of laryngeal dysfunction during exercise. This highlights the necessity for future work to determine whether targeted laryngeal intervention can improve dyspnea and exercise capacity in severe asthma [12]. Our report suggests that the scope of CLE extends beyond its original purpose, and findings from the study can have significant implications for the clinical decision-making process. As the focus of treatment for laryngeal anomalies shifts toward personalized medicine, CLE will become an even more prominent method for evaluating laryngeal dysfunction. Given this trend, the increase in diagnostic yield, and the minimal risk associated with this procedure, CPET with CLE will remain critical in the evaluation of laryngeal diseases and will provide safe and effective insight into the guiding management of individual patients. The follow-up study by Maat et al. concluded that relief of symptoms was experienced even in patients who were treated with information about the EILO diagnosis alone, although greater relief of symptoms and normalization of laryngeal function were significantly higher in a surgically treated group [10].

With further advances in technology, CLE will not only continue its current role in clinical practice but will also expand its scope as a minimally invasive advanced diagnostic tool. The involvement of a multidisciplinary approach makes the interpretation of findings and decision-making more reliable [11]. Select patients with a more complex medical history associated with combined aerodigestive pathologies may obtain more benefit from a decision-making standpoint. Since CLE has not been formally studied in randomized trials, further studies are needed to compare different diagnostic options to better define the appropriate indications and timing of CLE in order to better guide management.

## Conclusions

Continuous laryngoscopy during exercise is a safe and well-tolerated tool that can help diagnose various degrees of dynamic laryngeal dysfunction that constitutes a serious problem among children and adolescents, severely impairing their quality of life. Appropriate evaluation and diagnosis can help refine the next steps in the diagnostic process, prevent unnecessary diagnostic testing, and aid in tailoring the management of individual patients.
